# Preexcitation syndrome: experimental study on the electrocardiogram of antegradely conducting accessory pathway

**DOI:** 10.1186/s12872-018-0836-y

**Published:** 2018-05-21

**Authors:** Zhaolong Xu, Renguang Liu, Qinghua Chang, Changjun Li

**Affiliations:** 1 The Cardiovascular Institute of the First Affiliated Hospital of Jinzhou Medical University, Renmin Street, Jinzhou, 121000 Liaoning Province China; 2 Department of Respiration Medicine of the First Affiliated Hospital of Jinzhou Medical University, Renmin Street, Jinzhou, 121000 Liaoning Province China

**Keywords:** Preexcitation syndrome, Atrioventricular accessory pathway, Delta wave, Terminal QRS vector, Electrocardiogram

## Abstract

**Background:**

Preexcitation syndrome is characterized by a dominant delta wave on the baseline electrocardiogram (ECG), resulting from the change in QRS initial vector by the accessory pathway (AP). This study is to explore the effect of ventricular preexcitation on the QRS initial, maximal and terminal vector in an experimental rabbit with preexcitation syndrome induced by programmed electrical stimulation.

**Methods:**

Rabbits (*n* = 10) were randomized for the experimental model of ventricular preexcitation. Sensing and stimulating electrode catheters were placed in the high right atrium and along epicardial surface of atrioventricular groove of the left ventricular anterior wall, respectively. Programmed premature stimulation S_2_ was synchronized with P wave and utilized to stimulate the ventricle. The ECG recorded the electrical activity of the heart. As compared with the QRS complex during sinus rhythm, paced QRS was assessed regarding the initial, maximal and terminal vector. PS_2_ interval and PR interval were also measured and analyzed.

**Results:**

Preexcitation was successfully simulated by ventricular pacing in the rabbits, including (1) Complete preexcitation: PS_2_ interval was less than PR interval; the difference was more than or equal to 47.00 ± 7.53 ms. (2) Incomplete preexcitation: PS_2_ interval was less than PR interval; the difference was less than 47.00 ± 7.53 ms. (3) Incomplete latent preexcitation: PS_2_ interval was more than or equal to PR interval; the difference was less than or equal to 13.00 ± 3.50 ms. (4) Complete latent preexcitation: PS_2_ interval was more than or equal to PR interval; the difference was more than 13.00 ± 3.50 ms.

**Conclusions:**

The difference in the relative conduction velocity of the atrioventricular node versus the AP pathways determines the degree of preexcitation and different manifestation on ECG. The QRS terminal vector also reflects the ventricle preexcitation, indicating a valuable sign for the diagnosis of atypical or latent preexcitation.

## Background

Preexcitation syndrome is characterized by the electrocardiographic evidence of short PR interval as well as prolonged QRS containing a delta wave with the secondary ST-T change [1, 2]. A delta wave indicates that the part of the ventricle which is directly connected to accessory pathway (AP) is depolarized first. Preexcitation syndrome with antegrade AP conduction can be divided into overt and latent AP preexcitation based on the electrocardiogram (ECG) [3–5]. In patients with overt AP conduction, the ventricular preexcitation manifested as delta wave on surface ECG. While in patients with latent AP conduction, delta wave is invisible on surface ECG. In our recent study, we compared ECGs before and after ablation in preexcitation syndrome. This study confirmed that preexcitation affected not only the QRS initial vector but also the QRS maximum vector and the QRS terminal vector. Furthermore, we observed that two out of eight cases with latent AP preexcitation had no delta wave but QRS terminal vector changes on surface ECG, and we proposed “the incomplete latent preexcitation syndrome” [6, 7]. In the present study, in order to recognize the influence of the time difference of conduction through accessory pathway and normal pathway on the ECG of the preexcitation, we simulated preexcitation model by electrophysiological technique and changed the antegrade AP conduction, then further explored the effect of antegradely conducting AP on ECG characteristics.

## Methods

### Material

#### Ethical review

This study was carried out in strict accordance with the recommendations in the Guide for the Care and Use of Laboratory Animals of the National Institutes of Health. The protocol was approved by the Committee on the Ethics of Animal Experiments of the Jinzhou Medical University.

Ten healthy Japanese White Rabbits, weighing 2.0 ± 0.15 kg, were selected. All of the animals used in this study were housed in the Laboratory Animal Facility of our University.

### Preexcitation model

All the rabbits were anesthetized with urethane (1.2 g/kg) for induction. ECG monitor were then pasted to the animals. A left parasternal thoracotomy was performed, and the heart was exposed. The apex of one DAIG bipolar electrode (ST. JUDE company, America) was sutured to the epicardial surface of atrioventricular groove of left ventricular anterior wall, (Equivalent to the ventricular site of left anterior AP), and end of the electrode was kept outside through the cut, as ventricular pacing electrode. The other DIAG lead as atrial sensing electrode was placed at high right atrium via the internal jugular vein. The ends of the two leads were connected to the DF-4 electrophysiological stimulator (Oriental Electronic Company, Suzhou). Ventricular pacing was performed with voltage of twice the threshold of pulse amplitude, 2 ms of pulse width. We used a model ECG-6511 machine (Shanghai Medical Machine Company, China) to record surface ECG. Electrodes were placed on its arms and legs and right parasternal. The paper speed of ECG was 50 mm/s and the gain was 20 mm/mV. Programmed premature stimulation S_2_ synchronized P wave positively swept ventricle (PS_2_ interval: 0 → PJ interval, step length: 5 ms) to record surface ECG of chest lead, and the preexcitation model was simulated. Simultaneously, the ECGs were recorded. Programmed premature stimulation S_2_ synchronized P wave positively swept ventricle (PS_2_ interval: 0 → PJ interval, step length was 5 ms). The ECGs were recorded and preexcitation was simulated in the rabbit model. Cardioventricular pacing QRS complex by S_2_ after T wave was recorded which represents the QRS induced by complete ventricular preexcitation.

### Observed value

(1) The ventricular activation time via normal pathway (sinus PR interval); (2) The initial, maximal and terminal QRS vector through normal pathway (sinus QRS); (3) The ventricular activation time via AP (PS_2_ interval); (4) The initial, maximal and terminal QRS vector complete through AP (S_2_ pacing QRS after T wave); (5) The initial, maximal and terminal QRS vector with AP conduction (R_2_: Ventricular fusion wave formed by S_2_ and sinus conduction).

### The change of QRS vector

(1) Initial QRS vector change: the direction or amplitude of initial 20 ms QRS vector which was different from normal pathway conduction; (2) maximal QRS vector change: the amplitude or direction of maximal QRS amplitude which was different from normal pathway conduction. (3) Terminal QRS vector change: the direction or amplitude of terminal 20 ms QRS vector which was different from normal pathway conduction.

### Assessment of the degree of preexcitation

(1) complete preexcitation: R_2_ was absolutely equal to QRS complex through AP; (2) incomplete (typical) preexcitation: the initial vector of R_2_ was equal to QRS complex through AP, but the maximal and terminal vector of QRS alters between normal and AP conduction (ventricular fusion wave); (3) incomplete latent preexcitation: R_2_ was on behalf of ventricular fusion with terminal vector change; (4) complete latent preexcitation: R_2_ was absolutely equal to QRS complex via normal pathway (S_2_ encountered the effective refractory period of the ventricle).

## Results

### Preexcitation model by applying electrophysiological technique

**Fig. 1:**
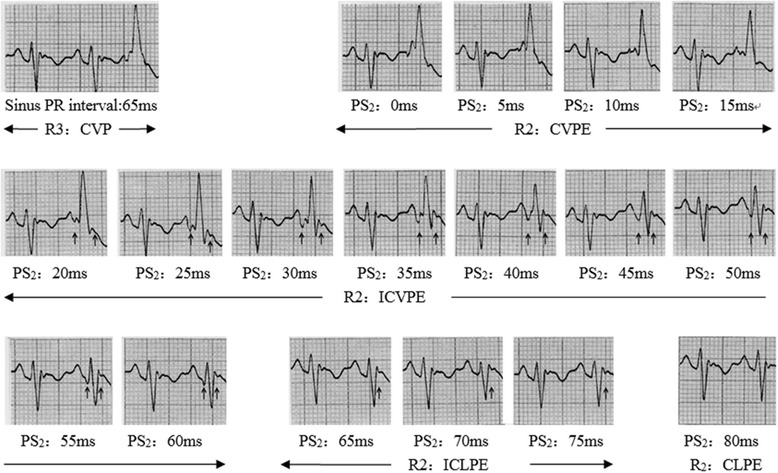
Four types of QRS complex recorded during ventricular pacing by synchronous pacing of PS_2_ and P wave simulating preexcitation of left anterior accessory pathway. Sinus PR interval represents the atrioventricular conduction time via normal pathway; PS_2_ represents atrioventricular conduction time via accessory pathway; R2 represents the different degrees of preexcitation; “↑” shows the changes of initial and terminal QRS vectors. CVP: complete ventricular pacing; CVPE: complete ventricular preexcitation; ICVPE: incomplete ventricular preexcitation; ICLPE: incomplete latent preexcitation; CLPE: complete latent preexcitation

Four kinds of different QRS waves were successfully observed on rabbit model of preexcitation, including complete preexcitation, incomplete (typical) preexcitation, incomplete latent preexcitation (terminal QRS vector change) and complete latent preexcitation (Fig. 1).

### The effect of the time difference of conduction through AP and normal pathway on the degree of ventricular preexcitation

The relationships between the degree of ventricular preexcitation and the atrioventricular (AV) conduction time via A-V nodal pathway (PR interval), AV conduction time via AP (PS_2_ interval), as well as the time difference of PR and PS_2_ interval were shown in Table 1. AP conduction was faster than AV node conduction (PS_2_ interval < PR interval), and R_2_ was on behalf of overt preexcitation. When the difference was ≥47.00 ± 7.53 ms, R_2_ was complete preexcitation, inversely, R_2_ was incomplete preexcitation. AP conduction is not faster than AV node conduction (PS_2_ interval ≥ PR interval), and R_2_ is latent preexcitation. When the difference was ≤13.00 ± 3.50 ms, R_2_ was incomplete latent preexcitation, inversely, R_2_ was complete latent preexcitation.

**Table 1:** The effect of PS_2_ interval, PR interval and its time difference on QRS complex

Model	PR interval ^a^(ms)	PS_2_ interval^b^/time difference between PS_2_ interval and PR interval^c^ (ms)			
		Complete ventricular preexcitation	Incomplete ventricular preexcitation	Incomplete latent preexcitation	Complete latent preexcitation
1	65	<25 / <-40	30~ 60 / -35~ − 5	65~ 85 / 0~ 20	>90 / >25
2	60	<15 / <-45	20~ 55 / -40~ − 5	60~ 75 / 0~ 15	>80 / >20
3	60	<25 / <-35	30~ 55 / -30~ − 5	60~ 70 / 0~ 10	>75 / >15
4	75	<15 / <-60	20~ 70 / -55~ − 5	75~ 85 / 0~ 10	>90 / >15
5	70	<5 / < -65	10~ 65 /−60~ − 5	70~ 85 / 0~ 15	>90 / >20
6	70	<20 / < -50	25~ 65 /−45~ − 5	70~ 80 / 0~ 10	>85 / >15
7	60	<20 / <-40	25~ 55 /−35~ − 5	60~ 75 / 0~ 15	>80 / >20
8	65	<15 / <-50	20~ 60 /−45~ − 5	65~ 75 / 0~ 10	>80 / >15
9	70	<20 / <-50	25~ 65 /−45~ − 5	70~ 85 / 0~ 15	>90 / >20
10	60	<15 / <-45	20~ 55 /− 40~ − 5	60~ 70 / 0~ 10	>75 / >15

Note:^a^indicates the time of conduction through normal pathway. ^b^indicates the time of conduction through accessory pathway. ^c^indicates the time difference of conduction through accessory pathway and normal pathway

## Discussion

In this study, programmed premature stimulation S_2_ synchronized P wave positively swept ventricle and ECG of antegradely conducting AP was successfully simulated in the rabbit model including complete preexcitation, incomplete (typical) preexcitation, incomplete latent preexcitation and complete latent preexcitation. This confirmed that the degree of preexcitation depended on the time difference of conduction through AP and normal pathway.

### Feasibility in this study

The canine model of preexcitation syndrome has been reported in clinical ECG study [8]. In our rabbit model, sensing electrode was placed to high right atrium (sense sinus P wave) and stimulating electrode was placed to atrioventricular groove of left ventricular anterior wall (simulate preexcitation through a left anterior AP). Programmed premature S_2_ which synchronized P wave (PS_2_ interval was equal to the conduction time through AP) performed stimulation and built the animal model of preexcitation syndrome in which sinus P wave can simultaneously conduct through normal pathway and left anterior AP. Various degree of preexcitation (complete preexcitation, incomplete preexcitation, incomplete latent preexcitation and complete latent preexcitation) were obtained during changing PS_2_ interval, confirming this study was a feasibility study. It can apply to the study of clinical ECG of preexcitation syndrome [9].

### The manifestation and influencing factor of antegrade conduction of AP in preexcitation syndrome

**Fig. 2:**
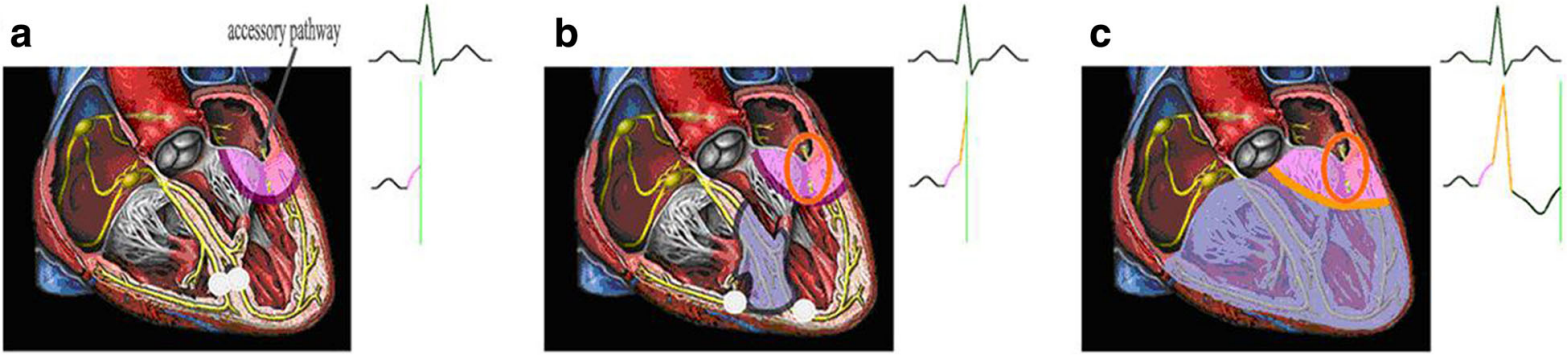
The delineation of the mechanism of preexcitation syndrome with overt accessory pathway. (**a**) The activation prematurely depolarizes the ventricle via accessory pathway which is faster than AV node, forming the delta wave. (**b**) The onset of the normal atrioventricular node conduction is the termination of the delta wave, but the accessory pathway conduction is continuous. (**c**) The activation simultaneously conducts through the accessory pathway and normal conduction system, fusing into the monophyletic ventricular fusion with delta wave in its initiation and the deformation in its termination

**Fig. 3:**
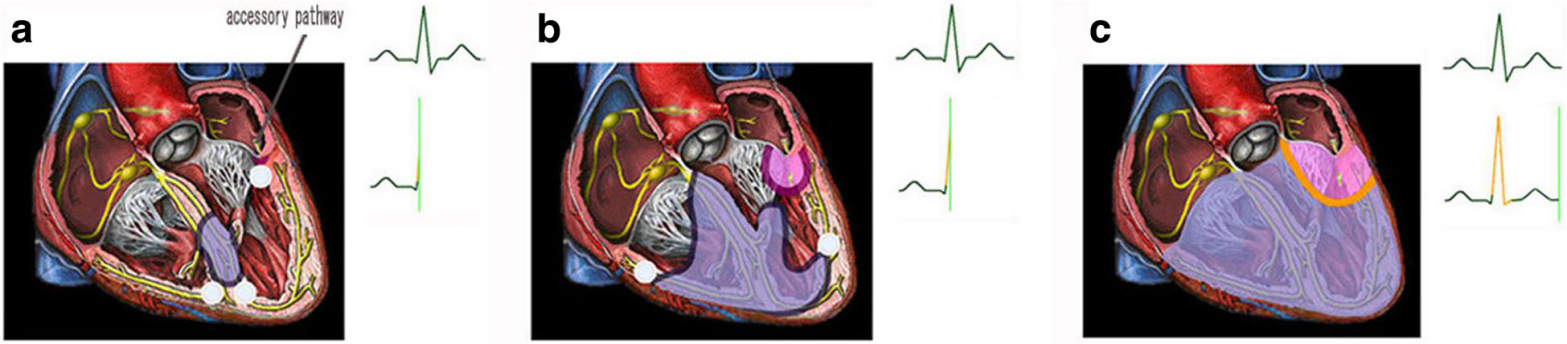
The delineation of the mechanism of incomplete latent preexcitation. (**a**) The atrioventricular node conduction is faster than or is equal to accessory pathway conduction, forming normal PR interval without delta wave. (**b**) The insertion of the ventricle is pre-excited by the activation conducted via the accessory pathway. (**c**) The activation simultaneously conducted through the accessory pathway and normal conduction system, fusing into the monophyletic ventricular fusion (mainly manifests the change of terminal QRS vector and morphology)

The preexcitation syndrome with AP capable of antegrade conduction could be divided into overt preexcitation and latent preexcitation. In latent preexcitation, the preexcitation is absent on the resting 12-lead ECG, which could be induced by transesophageal atrial pacing. Though there is no delta wave, the prevalence of arrhythmia and the risk of malignancy are same as the overt preexcitation [10–13]. In this study we successfully imitated the ECG manifestation of 4 type preexcitation including complete ventricular preexcitation, incomplete ventricular preexcitation, incomplete latent preexcitation, complete latent preexciation. The finding of this study further confirms that the ECG manifestation of antegrade conduction of AP depends on the relative time that the atrial impulse is conducted down the AP into ventricle as opposed to the normal pathway. (1) When the AP conduction is faster than the AV node conduction, the overt preexcitation is observed on surface ECG. The degree of preexcitation depends on the how much faster the atrial impulse is conducted down the AP into ventricle as opposed to the normal pathway. If the relative time that the atrial impulse is conducted down the AP into ventricle as opposed to the normal pathway is longer than the time that impulse transmits from the ventricle that is directly connected to AP to the normal pathway (the time of this group is 47.00 ± 7.53 ms), the atrial impulse can’t conduct into ventricle via the normal pathway, resulting in the complete ventricular preexcitation. If the relative time that the atrial impulse is conducted down the AP into ventricle as opposed to the normal pathway is no longer than the time that impulse transmits from the ventricle that is directly connected to AP to the normal pathway, the impulse can conduct into ventricle by the normal pathway and AP forming the monophyletic ventricular fusion, resulting in incomplete ventricular preexcitation (Fig. 2). (2) When the AP conduction is slower than the AV node conduction, the delta wave is absent. If the relative time that the atrial impulse is conducted down the AP into ventricle as opposed to the normal pathway is no longer than the time that impulse transmits from the normal pathway the ventricle that is directly connected to AP (the time of this group is 13.00 ± 3.50 ms), the AP is still able to produce premature ventricular depolarization in the part of the ventricle that is directly connected to the AP forming the ventricular fusion, resulting in the change of terminal QRS vector. During this time the incomplete latent preexcitation could be seen in the ECG (Fig. 3). If the relative time that the atrial impulse is conducted down the AP into ventricle as opposed to the normal pathway is longer or equal to the time that impulse transmits from the normal pathway the ventricle that is directly connected to AP, the impulse can’t conduct into ventricle via AP (encounter the effective refractory period of the ventricle), showing complete latent preexcitation in the ECG.

### The clinical significance of different degree of ventricle preexcitation

In this study the overt preexcitation was divided into complete and incomplete ventricular preexcitation based on the ECG manifestation. The clinical significance is as follows: as to the incomplete ventricular preexcitation which is the classical manifestation of preexcitation syndrome, it is easy to identify; as to the complete ventricular preexcitation, it is easy to misdiagnose resulting from lack of recognition. (1) When AP closes to the pacemaker (atrial premature beats), the intra-atrial conduction time is significantly shortened, resulting in the superimposition of P′ wave and QRS complex. During this time the PR interval is indistinguishable, and the premature atrial contraction is often misdiagnosed as premature ventricular contraction [14]. Furthermore, the polarity of initial QRS vector is identical to the delta wave, the finding of premature P′ wave is helpful for the diagnosis. (2) When the AV node conduction is obviously slower than AP conduction, such as first or third degree atrioventricular block (AVB) in normal pathway with complete ventricular preexcitation, the AVB in normal pathway is masked which is usually misdiagnosed in clinic [15–17]. During this time, the QRS complex is obviously widened and the PJ interval should be measured. The PJ interval prolongation provides that there is AVB in the normal pathway [15, 16, 18]. It is important for preoperative preparation and postoperative medical malpractice prevention to identify the presence of AVB according to ECG analysis before ablation.

In this study the latent preexcitation was divided into “incomplete latent preexcitation” and “complete latent preexcitation”. The clinical significance is as follows: the absence of delta wave is not means that the impulse can’t conduct down AP into ventricle. In patients with incomplete latent preexcitation syndrome, the delta wave is absent, but the impulse can conduct into ventricle via AP resulting in the change of terminal QRS vector [6]. The recognition of this new type preexcitation theoretically updates the recognition of delta wave. Meanwhile, it is helpful for the diagnosis of preexcitation syndrome mainly with a change of terminal QRS vector and the analysis of curative effect of bypass ablation [19–21]. However, it is not easy to observe the change of terminal QRS vector which could be found in comparison to the ECG during atrioventricular reentrant tachycardia or after ablation.

The multiple leads were not synchronously detected, and it had a certain influence on the accurate analysis of the terminal QRS vector. It will be helpful to analyze the relationship between the terminal QRS vector and the AP location if the multiple leads can be simulated. Besides, in previous study [22–24], researchers have observed the effects of ionic channel, gene, oxidative stress and inflammation on arrhythmias, and ventricular depolarization. Therefore, further studies are needed to assess whether these factors have influences on the electrocardiogram of antegradely conducting accessory pathway in future.

## Conclusions

(1) The ECG manifestation of antegrade conduction of AP depends on the relative time that the atrial impulse is conducted down the AP into ventricle as opposed to the normal pathway. The ECG includes manifest complete ventricular preexcitation, incomplete ventricular preexcitation, incomplete latent preexcitation, complete latent preexcitation according to the degree of preexcitation.

(2) In the preexcitation syndrome, the presence of a delta wave indicates that AV accessory pathway conduction is faster than AV node conduction; the antegrade conduction via AP impacts the QRS terminal vector changes; the QRS terminal vector changes indicate the activation download via AP and AV node and is helpful for the diagnosis of preexcitation, especially in patients with latent preexcitation syndrome without delta wave on ECG.

## Abbreviations

AP: Accessory pathway
AV: Atrioventricular
AVB: Atrioventricular block
ECG: Electrocardiogram
